# Supplementary material for: Modeling glioblastoma heterogeneity as a dynamic network of cell states
Source: Mol Syst Biol. 2021 Sep 16;17(9):e10105. doi: 10.15252/msb.202010105 (PMC8444284; doi:10.15252/msb.202010105)
Supplement: Supplementary file 5 — Source Data for Figure 3 [file MSB-17-e10105-s001.zip › Figure3A_sourcedata/GSEA_3065/nosigdb_state5.GseaPreranked.1622718435914/NB_KI_CLUSTER4.html]

Details for gene set NB\_KI\_CLUSTER4[GSEA]

|  || Dataset | state5 |
| Phenotype | NoPhenotypeAvailable |
| Upregulated in class | na\_neg |
| GeneSet | NB\_KI\_CLUSTER4 |
| Enrichment Score (ES) | -0.59682035 |
| Normalized Enrichment Score (NES) | -2.0400343 |
| Nominal p-value | 0.0 |
| FDR q-value | 0.0 |
| FWER p-Value | 0.0 |
Table: GSEA Results Summary

  

Fig 1: Enrichment plot: NB\_KI\_CLUSTER4      
 Profile of the Running ES Score & Positions of GeneSet Members on the Rank Ordered List

  

| PROBE | GENE SYMBOL | GENE\_TITLE | RANK IN GENE LIST | RANK METRIC SCORE | RUNNING ES | CORE ENRICHMENT || 1 | MEG3 |  |  | 22 | 0.783 | 0.0245 | No |
| 2 | MIAT |  |  | 58 | 0.619 | 0.0418 | No |
| 3 | LUC7L3 |  |  | 65 | 0.589 | 0.0614 | No |
| 4 | ATP6V0A1 |  |  | 205 | 0.436 | 0.0607 | No |
| 5 | SYT11 |  |  | 265 | 0.400 | 0.0679 | No |
| 6 | SEC11C |  |  | 287 | 0.388 | 0.0788 | No |
| 7 | CXADR |  |  | 303 | 0.381 | 0.0903 | No |
| 8 | CADM1 |  |  | 307 | 0.378 | 0.1029 | No |
| 9 | RIC3 |  |  | 308 | 0.378 | 0.1159 | No |
| 10 | ATP1B1 |  |  | 548 | 0.298 | 0.0993 | No |
| 11 | PHF14 |  |  | 837 | 0.234 | 0.0750 | No |
| 12 | RUFY3 |  |  | 839 | 0.234 | 0.0829 | No |
| 13 | NELFCD |  |  | 969 | 0.215 | 0.0758 | No |
| 14 | SLC38A1 |  |  | 992 | 0.212 | 0.0806 | No |
| 15 | MTSS1 |  |  | 1046 | 0.205 | 0.0817 | No |
| 16 | GLCCI1 |  |  | 1172 | 0.188 | 0.0741 | No |
| 17 | VAMP2 |  |  | 2075 | 0.107 | -0.0236 | No |
| 18 | ZNF704 |  |  | 2134 | 0.103 | -0.0266 | No |
| 19 | NSD3 |  |  | 2312 | 0.091 | -0.0433 | No |
| 20 | MDK |  |  | 2458 | 0.082 | -0.0568 | No |
| 21 | STMN3 |  |  | 2535 | 0.077 | -0.0627 | No |
| 22 | FXYD6 |  |  | 2847 | 0.060 | -0.0956 | No |
| 23 | GDAP1 |  |  | 3246 | 0.039 | -0.1390 | No |
| 24 | APLP1 |  |  | 3430 | 0.030 | -0.1586 | No |
| 25 | KIF3A |  |  | 3442 | 0.030 | -0.1588 | No |
| 26 | TSPAN13 |  |  | 3913 | 0.011 | -0.2112 | No |
| 27 | ZC3H13 |  |  | 3991 | 0.007 | -0.2196 | No |
| 28 | TRIM2 |  |  | 4188 | -0.001 | -0.2416 | No |
| 29 | RBFOX2 |  |  | 4204 | -0.001 | -0.2433 | No |
| 30 | CIRBP |  |  | 4617 | -0.019 | -0.2889 | No |
| 31 | KIF21A |  |  | 4843 | -0.027 | -0.3133 | No |
| 32 | GTF2I |  |  | 4961 | -0.032 | -0.3254 | No |
| 33 | NREP |  |  | 5261 | -0.043 | -0.3575 | No |
| 34 | COX6A1 |  |  | 5281 | -0.043 | -0.3582 | No |
| 35 | ATAT1 |  |  | 5330 | -0.046 | -0.3620 | No |
| 36 | STXBP1 |  |  | 5508 | -0.053 | -0.3801 | No |
| 37 | ATCAY |  |  | 5683 | -0.061 | -0.3975 | No |
| 38 | MYEF2 |  |  | 5684 | -0.062 | -0.3954 | No |
| 39 | ICA1 |  |  | 5718 | -0.063 | -0.3970 | No |
| 40 | ENO2 |  |  | 5895 | -0.069 | -0.4144 | No |
| 41 | TMEM14A |  |  | 6357 | -0.093 | -0.4630 | No |
| 42 | HSP90AB1 |  |  | 6611 | -0.109 | -0.4877 | No |
| 43 | CFAP20 |  |  | 7135 | -0.149 | -0.5414 | No |
| 44 | TCAF1 |  |  | 7202 | -0.155 | -0.5435 | No |
| 45 | GDI1 |  |  | 7381 | -0.179 | -0.5573 | No |
| 46 | PRKAR2B |  |  | 7651 | -0.211 | -0.5803 | No |
| 47 | MAP1B |  |  | 7666 | -0.213 | -0.5746 | No |
| 48 | HDAC2 |  |  | 7865 | -0.247 | -0.5883 | Yes |
| 49 | OCIAD2 |  |  | 7875 | -0.249 | -0.5808 | Yes |
| 50 | RBP1 |  |  | 7877 | -0.250 | -0.5723 | Yes |
| 51 | SOX4 |  |  | 7958 | -0.268 | -0.5721 | Yes |
| 52 | CENPV |  |  | 8000 | -0.276 | -0.5672 | Yes |
| 53 | TERF2IP |  |  | 8065 | -0.289 | -0.5645 | Yes |
| 54 | ATP6V0E2 |  |  | 8103 | -0.298 | -0.5584 | Yes |
| 55 | NLRP1 |  |  | 8106 | -0.298 | -0.5483 | Yes |
| 56 | QDPR |  |  | 8112 | -0.299 | -0.5386 | Yes |
| 57 | TTC3 |  |  | 8127 | -0.303 | -0.5298 | Yes |
| 58 | CDK6 |  |  | 8156 | -0.310 | -0.5223 | Yes |
| 59 | MCRIP1 |  |  | 8164 | -0.314 | -0.5123 | Yes |
| 60 | CBX1 |  |  | 8222 | -0.331 | -0.5073 | Yes |
| 61 | TUBB2B |  |  | 8224 | -0.332 | -0.4960 | Yes |
| 62 | CCND1 |  |  | 8346 | -0.368 | -0.4969 | Yes |
| 63 | TUBB2A |  |  | 8364 | -0.376 | -0.4859 | Yes |
| 64 | C3orf14 |  |  | 8424 | -0.396 | -0.4789 | Yes |
| 65 | CKB |  |  | 8448 | -0.407 | -0.4675 | Yes |
| 66 | MYL6B |  |  | 8477 | -0.423 | -0.4561 | Yes |
| 67 | SNRPN |  |  | 8504 | -0.436 | -0.4440 | Yes |
| 68 | NME1 |  |  | 8573 | -0.471 | -0.4355 | Yes |
| 69 | PEG10 |  |  | 8628 | -0.510 | -0.4240 | Yes |
| 70 | YWHAE |  |  | 8629 | -0.512 | -0.4064 | Yes |
| 71 | JPT1 |  |  | 8647 | -0.523 | -0.3903 | Yes |
| 72 | DPYSL3 |  |  | 8692 | -0.554 | -0.3762 | Yes |
| 73 | MLLT11 |  |  | 8695 | -0.555 | -0.3573 | Yes |
| 74 | UCHL1 |  |  | 8700 | -0.565 | -0.3384 | Yes |
| 75 | FSCN1 |  |  | 8710 | -0.573 | -0.3197 | Yes |
| 76 | YWHAH |  |  | 8726 | -0.587 | -0.3012 | Yes |
| 77 | YWHAQ |  |  | 8730 | -0.592 | -0.2812 | Yes |
| 78 | CALM2 |  |  | 8744 | -0.600 | -0.2620 | Yes |
| 79 | CALM1 |  |  | 8748 | -0.609 | -0.2414 | Yes |
| 80 | BEX3 |  |  | 8759 | -0.619 | -0.2212 | Yes |
| 81 | TUBB |  |  | 8763 | -0.621 | -0.2002 | Yes |
| 82 | STMN1 |  |  | 8783 | -0.638 | -0.1804 | Yes |
| 83 | GNAS |  |  | 8822 | -0.674 | -0.1615 | Yes |
| 84 | H3F3B |  |  | 8846 | -0.705 | -0.1398 | Yes |
| 85 | SUMO2 |  |  | 8852 | -0.712 | -0.1159 | Yes |
| 86 | MARCKSL1 |  |  | 8857 | -0.716 | -0.0917 | Yes |
| 87 | H3F3A |  |  | 8867 | -0.728 | -0.0677 | Yes |
| 88 | TUBA1A |  |  | 8883 | -0.751 | -0.0435 | Yes |
| 89 | PEBP1 |  |  | 8896 | -0.769 | -0.0184 | Yes |
| 90 | PTMS |  |  | 8921 | -0.820 | 0.0071 | Yes |
Table: GSEA details [plain text format]

  

Fig 2: NB\_KI\_CLUSTER4: Random ES distribution      
 Gene set null distribution of ES for **NB\_KI\_CLUSTER4**
